# Disease-Related Malnutrition in Pediatric Patients with Chronic Disease: A Developing Country Perspective

**DOI:** 10.1016/j.cdnut.2022.100021

**Published:** 2022-12-23

**Authors:** Mirari Prasadajudio, Yoga Devaera, Noormanto Noormanto, Rahmat B. Kuswiyanto, Bambang Sudarmanto, Murti Andriastuti, I Gusti Lanang Sidiartha, Nova L. Sitorus, Ray W. Basrowi

**Affiliations:** 1 Department of Child Health, Hermina Hospital Kemayoran, Jakarta, Indonesia; 2 Department of Child Health, Medical Faculty, Universitas Indonesia, Cipto Mangunkusumo Hospital, Jakarta, Indonesia; 3 Department of Child Health, Sardjito Hospital, Faculty of Medicine, Universitas Gadjah Mada, Yogyakarta, Indonesia; 4 Department of Child Health, Hasan Sadikin Hospital, Faculty of Medicine, Universitas Padjajaran, Bandung, Indonesia; 5 Department of Child Health, Kariadi Hospital, Faculty of Medicine, Universitas Diponegoro, Semarang, Indonesia; 6 Department of Child Health, Sanglah Hospital, Faculty of Medicine, Universitas Udayana, Denpasar, Indonesia; 7 Danone Specialized Nutrition, Jakarta, Indonesia

**Keywords:** children, malnutrition, chronic disease, disease-related malnutrition, developing country

## Abstract

Malnutrition is widely known to affect growth in children. There are many studies focusing on malnutrition globally in relation to limited food access; however, there is only limited research on disease-related malnutrition, especially in chronic conditions and particularly in developing countries. This study aims to review articles on the measurement of malnutrition in pediatric chronic disease, especially in developing countries where there are resource limitations in identifying nutritional status in pediatric chronic disease with complex conditions. This state-of-the-art narrative review was conducted through search of literatures through 2 databases, and identified 31 eligible articles published from 1990 to 2021. This study found no uniformity in malnutrition definitions and no consensus regarding screening tools for the identification of the malnutrition risk in these children. In developing countries where resources are limited, instead of focusing on finding the best tools to identify the malnutrition risk, the approach should be directed toward developing systems that work best according to capacity and allow for a combination of anthropometry assessment, clinical evaluation, and observation of feeding access and tolerance on a regular basis.

## Introduction

Pediatric malnutrition is an ongoing burden in developing countries, particularly in Southern Asia, as compared with Latin America and the northern and western regions of Africa. It is globally known to be the most crucial risk factor for morbidity and mortality [1]. Approximately 1.9 billion people in the Asia and Pacific region have limited access to a healthy diet, and the need to improve the diet quality, especially in children, is critical [2]. However, when we assess the pathogenesis and pathophysiology of malnutrition, there should be a differentiation between nondisease-related malnutrition, also known as nonillness-related malnutrition, and disease-related or illness-related malnutrition [3].

The first type of malnutrition occurs in relation to environmental factors, such as limited food access or decreased dietary intake, which results in an imbalance relative to nutritional needs, thereby affecting growth, development, and other health aspects. Disease-related malnutrition may progress to acute or chronic disease and occurs because of an energy imbalance due to increased catabolism, decreased appetite, or increased energy losses, which also have detrimental effects on the outcomes [3,4].

Studies have clearly observed that disease-related malnutrition is associated with an increase in the length of stay of hospitalized children and additional financial burdens. A study in the Netherlands noted the annual medical costs for the country, estimated at €80 million for disease-related malnutrition, which equals 5.5% of the total hospital costs for these hospitalized children [5]. Disease-related malnutrition, especially in pediatric chronic disease, requires long-term nutrition support to prevent long-term growth faltering and impaired development and also to prevent poor health-related quality of life [6,7].

The causes of malnutrition in children with chronic disease are multifactorial and are associated with the underlying disease; thus, careful assessment and management are required for chronic disease.

This study observed the most common chronic diseases in children, such as congenital heart disease (CHD), chronic kidney disease (CKD), chronic liver disease (CLD), and malignancy, in which their nutritional assessment may be complicated with preceding conditions such as feeding intolerance, water retention, peripheral edema, electrolyte disturbance, tumor masses, and decreases in bone density and fat mass [[8], [9], [10], [11]]. Indonesia, like other developing countries, also faces malnutrition associated with infectious diseases, such as diarrhea, AIDS, tuberculosis, and parasite infections. However, it is also observed that nutritional deficiency is known to be a predisposing factor for poor immune response, which makes children more susceptible to infection [12]. The 3 factors undernutrition, infectious diseases, and immune systems form a simple cycle and make their roles interlinked [13].

Our research focused on chronic malnutrition in children with chronic diseases as mentioned above, which has received less attention in developing countries than that on malnutrition caused by infectious diseases. The combination of anthropometry assessment, clinical evaluation, observation on feeding tolerance, and biochemistry analysis should be conducted on a regular basis to ensure adequate nutrition. Lack of uniform definitions, heterogenous screening practices, and the failure to recognize nutrition as part of crucial patient care, however, have contributed to underrecognition of the prevalence of malnutrition in relation to its outcomes [3,4]. In developing countries, assessment also depends on the availability of resources and regular monitoring to achieve good outcomes [11].

The initial concept of the study was derived from an expert meeting that consisted of 6 child health professionals, who contributed input and presented current challenges in managing malnutrition in chronically ill children. The purpose of this study was to review articles available on the measurement of malnutrition in pediatric chronic disease, especially in developing countries where resource limitations restrict identification of the nutritional status in pediatric chronic disease with complex conditions.

## Methods

This is a state-of-the-art narrative review conducted through search of literatures on the measurement of malnutrition in pediatric chronic diseases. There are 5 domains included in the overall setup of the definition: anthropometrics, screening tools, etiology, chronicity, and impact on functional status and its management. These domains were used to evaluate and explore the implications of malnutrition in chronic disease.

To identify articles published on disease-related malnutrition in pediatric chronic disease, we conducted keyword searches in PubMed and PubMed Central (PMC). The literature search was conducted from July 2021 to August 2021.

For PubMed, we applied the following MeSH terms: “Malnutrition”[Mesh] OR “Child Nutrition Disorders”[Mesh] OR “Infant Nutrition Disorders”[Mesh]. We limited our search to papers published in or after the year 1990 to achieve certain relevancy to socioeconomics, available diagnosis tools, and management. For PMC, we used the following search terms on papers published from 1990 to 2021 for all MEDLINE journals: “pediatrics”[All Fields] AND “malnutrition”[All Fields] AND “chronic disease”[All Fields]. We did not limit the last search with MeSH terms to ensure that we have included recent papers that were not yet indexed, and may not miss any synonym in terminology that maybe relevant to our research questions.

The following inclusion criteria were applied during our search of literatures:

• Study site: Studies conducted in various countries at any level can be outpatient or inpatient settings.
• Design: Randomized and nonrandomized controlled trials and observational studies.
• Outcome: Disease-related malnutrition in children at any age between 0 and 18 y with chronic disease.
• Relevance: Studies published that addressed any factors identified in the ASPEN framework.

For observational studies, we included variables depending on the available data and statistical methods. Statistical associations reported are considered significant, with *P* values less than or equal to 0.05 and 95% CIs.

**FIGURE 1:**
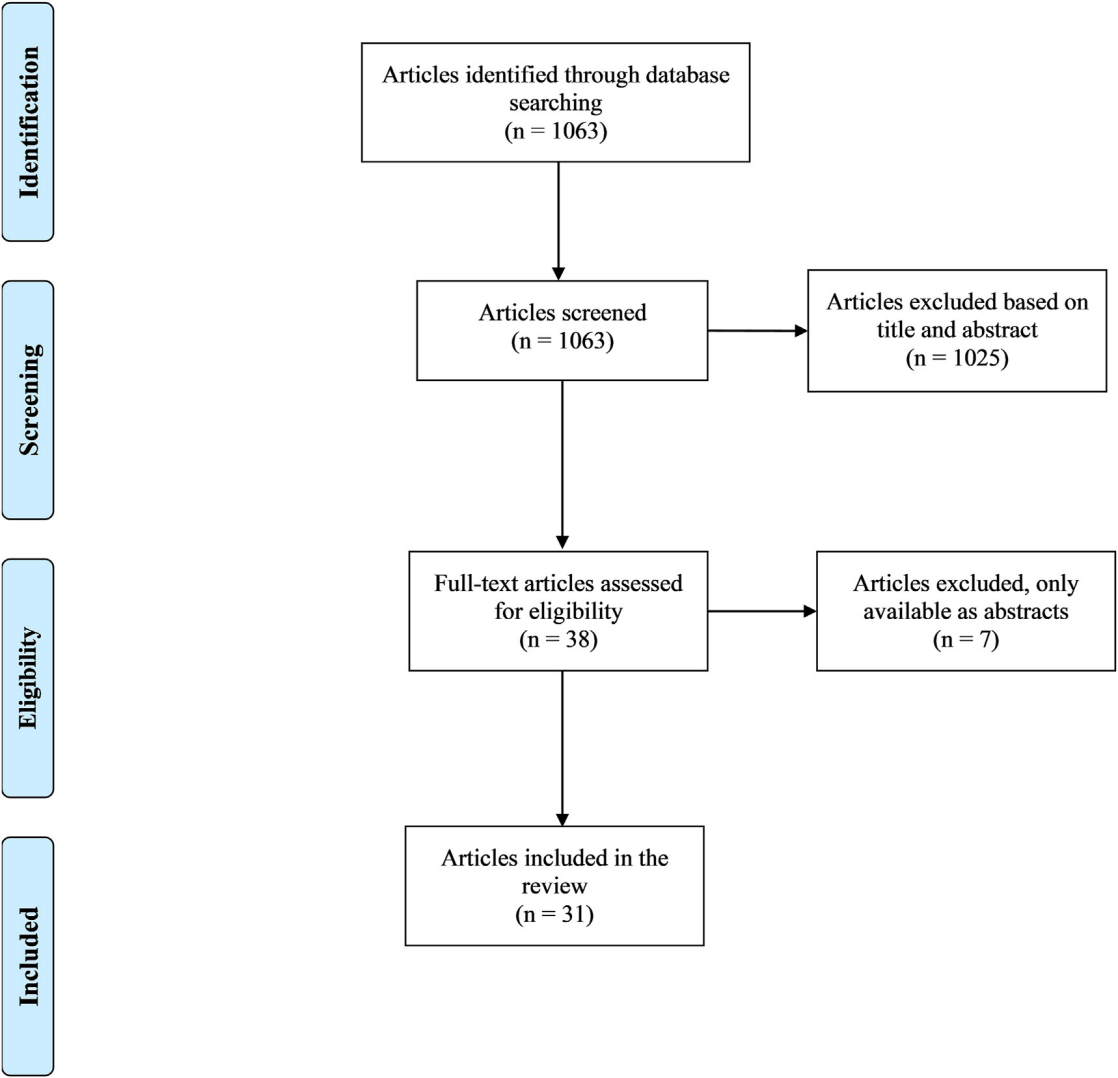
PRISMA flow diagram detailing article selection process.

Articles not published in English, published before 1990, and articles not available for free in full texts were excluded from this narrative review. The PRISMA flow diagram containing details of article selection process is presented in Figure 1.

## Results

We acquired a total of 1063 articles from searches using the aforementioned search terms. Of the 1063 articles found, we excluded 1025 articles after screening based on the titles and abstracts, resulting in 38 eligible articles. Another 7 articles were further excluded because of the fact that these articles were available only as abstracts, resulting in a total of 31 full-text articles reviewed.

These articles were reviewed using the aforementioned 5 domains to establish a better framework for the understanding of disease-related malnutrition (Table 1). As malnutrition can occur in nondisease-related or disease-related forms, the term malnutrition in this study from now on will refer only to disease-related malnutrition.

**TABLE 1:** Five domains for review and some questions to address

Domain	Questions to address
A. Anthropometric variables	• What anthropometric variables should be measured in assessing the status of pediatric patients with disease-related malnutrition? • Should SD, percentiles, or Z-scores be used to classify nutrition status? • What assessment tools should be used in conditions of abnormalities in body composition?
B. Screening tools	• What screening tools can be used to detect malnutrition risk for growth evaluation?
C. Chronicity	• What is the cut-off period for chronicity?
D. Etiology of malnutrition	• What is the impact of underlying disease on nutrition status?
E. Impact of malnutrition on functional status and management	• What are the outcomes resulting from malnutrition? • What is the suggested management?

SD, standard deviations.

### Anthropometric variables

Pediatric patients with chronic disease have been known to be an increased risk of malnutrition, which leads to poor outcomes. Anthropometric assessment can be challenging in this group when the disease and nutritional status have a reciprocal effect. Additionally, malnutrition in chronic disease is also complicated by changes in the body composition such fat mass, fat-free mass, and total-body water [14]. Regular evaluation is crucial at the point of admission and during the hospital stay, discharge, or ambulatory settings to ensure adequate nutrition in these patients.

The American Society of Parenteral and Enteral Nutrition (ASPEN), under the WHO guidelines, recommended that anthropometric measurements should be expressed as Z-scores. Mild malnutrition was defined with Z-scores of −1 to −1.9, whereas moderate malnutrition was defined with a Z-score between −2 and −2.9 and severe malnutrition was defined with a Z-score of <−3. Furthermore, the consensus also endorsed the use of weight-for-height (WFH), BMI, or height-for-age Z-score (HAZ) in the WHO’s child growth standards from birth to the age of 2 y, and the CDC growth charts for children of ages 2–20 y [15]. Some countries may also use a local chart or continue following up the growth using WHO charts. In critically ill patients such as those admitted to pediatric intensive care units, patients must undergo a nutritional assessment at admission and are re-evaluated weekly throughout their stay. BMI or weight-for-age Z-scores (WAZs) are also recommended to be used when patients are < 2 y of age, and the head circumference must be documented when they are less than 36 mo of age [16].

Adjusted measurements and careful interpretation are needed for malnourished children with chronic disease, because the nutrition status is compromised by complex conditions, such as decreases in bone density, organomegaly, or water retention. This review observed studies on chronic diseases in children that are mostly known to be related to challenges in malnutrition due to altered body composition: chronic heart, kidney, liver disease, and malignancy.

In CHD, age was found to be a significant indicator of acute and chronic malnutrition; acute malnutrition was the most prevalent type in infants, while chronic malnutrition was mostly found in toddlers [17]. A study regarding the nutritional status in neonates with CHD undergoing cardiac surgery stated that the measurement of WAZ in assessing malnutrition in this age group is sufficient for predicting the need of prolonged respiratory support and the risk of mortality [18]. Another study of 2088 children under 5 y of age with CHD also confirmed the usefulness of WAZ measurements accompanied by HAZ in detecting preoperative malnutrition [19]. A smaller analytical study observed the following parameters: weight-for-age, height/length, head circumference, mid-arm circumference, and triceps and subscapular skinfold thickness in 50 children aged 0–17 y with CHD prior to cardiac surgery. The study concluded that children with CHD experienced growth retardation (*P* < 0.001, t-test). Although the study did not specify the cut-off age for measuring the head circumference, it observed that all of the above measurements, except the head circumference, differed significantly from the healthy standard [20].

CKD is known to be associated with muscle deficit and growth failure. One study suggested that, in children and adolescents with CKD, lean mass (LM) and fat mass (FM) need to be characterized using dual-energy X-ray absorptiometry (DXA) scans [21]. Notwithstanding, Rashid et al. [22] pointed out some limitations in the use of DXA to assess body composition. When there is a fluid overload, an overestimation of LM can occur. The study deduced that BMI measurements may not be apparent in CKD with abnormalities in body composition; instead, DXA can be performed with careful interpretation.

Children with CLD are also prone to difficulties in anthropometric measurements due to ascites, peripheral edema, or enlarged liver. One study recommended mid-arm circumference, mid-arm muscle circumference, and triceps skinfold thickness (TSFT) [23].

The evaluation of nutritional status in children with cancer is also crucial, but weight measurements, as a base, can be problematic, especially in those with advanced disease. Use of DXA has been recommended to assist anthropometry measurements; however, they are not commonly available in developing countries. In cases where DXA is not available, arm anthropometry can be performed. A study showed that the mid-upper arm circumference (MUAC) corresponded well with lean body mass as measured using DXA, but skinfold thickness was a poor estimate of FM. However, in solid tumors, the interpretation was found to be limited and needs further study [24]. A recent study observing pediatric cases with solid tumors showed a reduction in fat-free mass. The study claimed that arm-muscle measurement showed a positive correlation with fat-free mass measured by bioimpedance analysis and deuterium oxide [25].

### Screening tools

The assessment of the malnutrition risk is crucial in chronic disease so that growth can be monitored regularly and adverse outcome can be prevented in both inpatient and outpatient settings. There are different types of known pediatric screening tools that have been established and studied in pediatrics: STRONGkids [26], screening tool for the assessment of malnutrition in pediatrics (STAMP) [27], pediatric Yorkhill malnutrition score (PYMS) [28], nutrition risk score (NRS) [28], pediatric nutritional risk score (PNRS) [29], Pediatric Nutrition Screening tool (PNST) [29], and subjective global nutrition assessment (SGNA) [30]. Because there are several nutritional screening tools used to determine the malnutrition risk in children, a systematic review was performed to review the performance of 7 nutrition screening tools in terms of validity and reproducibility: PYMS, iPYMS, PeDiSMART, PNR, STAMP, PMST, and STRONGkids. The study recommended the use of PYMS in clinical practice, while in hospital settings, STAMP and iPMYS rules were preferable; thus, careful usage should be considered according to each clinical setting [31].

To date, there is much research available that focuses on these screening tools in assessing the malnutrition risk in general and comparing one tool with another; however, there is limited research specifically on preferable tools for screening malnutrition in pediatric patients with chronic disease.

Only 3 papers were found that observed malnutrition risk in pediatric patients with chronic disease during the entry point of admission, assessed using STAMP, STRONGkids, PYMS, or PNST. All these studies were conducted in different countries: Italy, Canada, and the UK. They also used the same prospective observational design study, but with different populations and points of outcome (Table 2).

**TABLE 2:** Overview of screening tools in identifying malnutrition risk and related problems in chronic disease

Study	Year	Study design	Population	Intervention (screening tools)	Outcome/summary points	Statistical test
Spagnuolo et al. [32]	2013	Prosepective observational	1–18 y old inpatients (n = 144)	STRONGkids	STRONGkids is recommended with high sensitivity but not specificity.	Increased risk of malnutrition:
						71% sensitivity (95% CI: 48–89), 53% specificity (95% CI: 43–63)
						(LR 1.5; p = 0.032) based on anthropometrics.
Lara-pompa et al. [33]	2020	Prospective observational	5–18 y old inpatients (n = 152)	STAMP	STAMP and STRONGkids are good tools for screening malnutrition on admission, whereas PYMS is the best tool for identifying pediatric cases with adverse clinical outcomes.	Increased LOS
				STRONGKids	Increased LOS and outcomes of complications were provided as complementary information for malnutrition risk.	PYMS: 49% sensitivity, 81% specificity;
				PYMS		STRONGkids: 46% sensitivity, 68% specificity;
						STAMP: 34% sensitivity, 86% specificity.
						Outcome of complications
						PYMS: 55% sensitivity, 83% specificity;
						STRONGkids: 52% sensitivity, 69% specificity; STAMP:
						33% sensitivity, 86% specificity
Carter et al. [34]	2020	Prospective observational	1 mo–17y old Inpatients (n = 165)	Strongkids PNST	PNST is not a better screening tool than STRONGkids unless adapted to each population by adjusting the cut-off values for nutrition risk as compared with SGNA.	Increased risk of malnutrition
						PNST: 88% sensitivity, 78% specificity; Cohen’s kappa (κ), 0.658 (*P* < 0.001);
						STRONGkids: 94% sensitivity, 44% specificity; κ, 0.38 (*P* = 0.009)
						ROC curve for PNST: 0.819 (0.745–0.894), *P* < 0.001.
						ROC curve for STRONGkids:
						0.809 (0.723–0.894), *P* < 0.001

CI, confidence interval; LOS, length of stay; LR, likelihood ratio; PNST, pediatric nutrition screening tool; PYMS, pediatric Yorkhill malnutrition score; ROC, receiver operating characteristic; SGNA, subjective global nutritrion assessment; STAMP, screening tool for the assessment of malnutrition in pediatrics; STRONGkids, screening tool risk on nutritional status and growth for kids.

An Italian study conducted by Spagnuolo et al. [32] collected data on hospitalized children between 1 and 18 y of age who were admitted to 12 different hospitals and had known underlying chronic diseases associated with infectious, gastrointestinal, respiratory, metabolic, neurological, oncological, and cardiac diseases, as well as trauma and surgical cases, with the exclusion of patients in intensive care. The malnutrition risk was assessed using STRONGkids as the screening tool.

On the other hand, Lara-pompa et al. [33] included children aged 5–18 y hospitalized in a tertiary London hospital with the duration of stay longer than 3 d as the cut-off point for chronic conditions. PYMS, STAMP, and STRONGkids were chosen as the tools to determine the risk of impaired growth and nutrition at admission. Although the study did not specify the underlying diseases, their patients included those with restricted diets and fluids, under steroid medication, undergoing enteral/parenteral feeding, who received prior dietetic advice, who were wheelchair users, and undergoing surgery.

Carter et al. [34] studied children between 1 mo and 17 y of age who were admitted to surgery and medicine units in a Canadian hospital, and excluded those with a length of stay less than 24 hours; chronicity was decided by the condition of the disease. Unlike the previous 2 studies, Carter et al. used the SGNA as a reference standard to determine the concurrent validity of PNST and STRONGkids as screening tools in the accurate identification of malnourished children, especially in patients with neurological, cardiovascular, gastrointestinal, metabolic, oncological, and nephrological diseases. The studies are summarized in Table 2.

Studies by Spagnuolo et al. [32] and Carter et al. [34] both agree that STRONGkids is highly sensitive in identifying malnutrition risk in children with chronic disease admitted to hospital, but with low specificity. The sensitivities were 71% and 94%, whereas the specificities were 53% and 44% for both studies, respectively. Lara-pompa et al. [33] observed that STRONGkids showed a low sensitivity but high specificity for the outcome of the length of stay and adverse clinical outcomes. The study concluded that STAMP and STRONGKids were the best at detecting malnutrition on admission, whereas PYMS was helpful in identifying children with adverse clinical outcomes.

Both of the studies that used 2 or more screening tools concurred that agreement between the diagnostic parameters was poor. One screening tool is not superior to others, although one may detect certain things the others did not, such as the length of stay or adverse outcomes.

However, some health centers still hold SGNA valid in verifying the actual nutrition status of a pediatric population, and thus used this particular tool as their reference [[34], [35], [36]]. One of the pioneer nutritional status assessments is PNRS, which analyzed 3 nutritional risk factors—food intake, pain, and disease severity—and thereby concluded that these 3 factors were the most important in predicting weight loss during hospitalization. Although not widely used as compared with the other tools, it has also been found useful in detecting the malnutrition risk in general [15,29].

In an ambulatory setting, a study in the Netherlands evaluated chronically ill children attending special schools, using STRONGkids for profile screening in this group, and concluded that identifying the nutritional status in children with chronic disease is important to prevent lower health status, especially in young children with chronic medication usage [37].

### Chronicity

Malnutrition can be categorized as acute (less than 3 mo in duration) or chronic (duration of 3 mo or more). Acute malnutrition is hallmarked by a decrease in the patient’s WFH, whereas chronic malnutrition may be shown by a decreased height velocity (stunting), which may be characterized by a HAZ of less than −2 [3]. Chronic malnutrition may be irreversible and occurs prior to hospital admission or during hospitalization (hospital-acquired malnutrition).

### Etiology of malnutrition

In disease-related malnutrition, nutrient deficiency can be associated with inflammation or cannot be associated. Inflammation can also be regarded as cachexia characterized by the loss of muscle mass, with or without the depletion of FM, whereas noninflammation may be due to disease-triggered malnutrition, such as dysphagia from upper digestive obstruction or neurologic disorders [38].

Children with CHD experienced a hypermetabolic state with a 30% increase in resting energy expenditure [17]. Malnutrition tends to be the most prominent between 1 mo and 1 y of age due to increased metabolic demand, which puts these children in catabolic stress [19]. CKD may lead to a reduced nutrient intake secondary to toxin accumulation, changes in satiety regulatory hormones, inflammation, the effects of peritoneal dialysis on one’s sense of fullness, nutrient depletion associated with dialysis, anemia in relation to hyporesponsiveness to erythropoietin and iron, alterations in growth hormones, and protein-energy wasting [14,39].

On the other hand, CLD occurs as a consequence of the decrease in consumption, increased energy requirements, or fat malabsorption from cholestasis [40]. Malnutrition in patients with cancer was speculated to be associated with proinflammatory cytokines (IL-1α, IL-1β, IL-6) expressed by tumor tissue, immune and stroma cells, TNFα, and INFγ, along with other mediators affecting food intake and energy expenditure, thereby leading to the clinical syndrome of cancer cachexia. Moreover, increased nutrient requirements, therapy-induced toxicity resulting in gastrointestinal dysfunction, and changes in metabolic and hormonal states play some roles in the progress of malnutrition in this group [41].

### Impact of malnutrition on functional status and its management

In developing countries with limited resource for treatment or surgical intervention, the prevalence and severity of malnutrition, growth failure, and pulmonary hypertension in children with CHD increased dramatically [42]. Moreover, preoperative malnutrition in children with CHD was found to be associated with adverse outcomes after pediatric surgery, including death, cardiac arrest, infection, increased length of ICU and hospital stay, and longer duration of mechanical ventilation [19].

However, if surgical intervention was incorporated early in the first 30 d of life, the outcome was observed to be good. A study showed that the total energy expenditure in these infants did not differ significantly compared with age-matched healthy infants at the follow-up ages of 3 and 12 mo. The study also concluded that dietary reference intake may be used as a reference to estimate the energy intake in children with CHD who have had surgical intervention [43]. The recommendations were [89 × weight (kg) − 100] + 175 kcal/d for 0–3 mo of age and [89 × weight (kg) − 100] + 22 kcal/d for 7–12 mo of age [44].

For newborns with CHD, human milk is believed to be better tolerated, promotes intake and growth, and is associated with less postoperative complications. It can be given through oral feeds or enteral feeding tubes [45]. Formulas with a higher calorie concentration (above 0.67 kcal/mL) can be given as an option when required. Feed intolerance can be affected by the caloric density of feeds, and transitioning to hydrolyzed or elemental formula may help improve feed tolerance.

Under perioperative conditions in particular, early initiation of enteral nutrition is shown to be related to better outcomes of wound healing, less gastrointestinal dysfunction, and reduced muscle wasting. Perioperative EN is suggested to be high in calorie content and should be adjusted to individual needs or water restriction if required. In the immediate postoperative period (0–3 d), nutrition required is ∼35–65 kcals/kg/d since REE in this condition is considerably reduced (Table 3) [46].

**TABLE 3:** Overview of nutritional assessment in pediatric chronic disease

Chronic disease	Anthropometric measurement	Problems in nutritional assessment	Etiology of malnutrition	Management and monitoring
Congenital heart disease	Weight-for-age (newborn)	• Fluid overload • Feeding intolerance • Water restriction	Hypermetabolic state	Diet
	Height-for-age (newborn)		Catabolic stress	Infants: breastmilk
	Weight-for-length			Perioperative: high-calorie formula
	TSFT			Immediate postoperative (0–3 d): nutrition 35–65/kg/d with reduction of REE
	MUAC			
CKD	LM	• Muscle deficit • Water retention • Overestimation of lean mass •Underestimation of fat and protein mass	Toxin accumulation	Diet
	FM		Reduced satiety	Infants: breastmilk
	BMI		Nutrient depletion	Regular formula
	Skinfold thickness		Protein-energy wasting	Lower protein diet of 0.6–0.8 g/kg/d
	MUAC		Iron deficiency anemia	Restricted potassium: 40–120 mg/kg/d for infants, 30–40mg/kg/d for older children.
	DXA			Restricted sodium: 1500–2400 mg/d
	Isotope dilution			Periodic check-up: 3–4 mo
				Regular urine analysis
CLD	TSFT	• Ascites • Edema • Enlarged liver	Decreased in consumption	Diet
	Arm anthropometry		Increased energy requirements	Infants: breastmilk
	BMI		Fat malabsorption	Formula with <75% MCT
	DXA			Carbohydrate: 6–8 g/kg/d
				Protein: 2.5–3 g/kg/d
				Fat: 5–6 g/kg/d
Cancer	MUAC	• Tumor mass • Hydration (during chemotherapy) • Wasting of fat and skeletal muscle	Cancer cachexia	Diet
	TSFT		Increased nutrient requirements	Infants: breastmilk
	DXA		Therapy-induced toxicity	High-calorie formulas
				Individualized nutritional care plan (based on ideal body weight, BMI, and estimated energy needs)
				Screening nutrition status: every 4 wk

BMI, body mass index; CKD, chronic kidney disease; DXA, dual-energy X-ray absorptiometry; FM, fat mass; LM, lean mass; MCT, medium-chain triglyceride; MUAC, middle upper-arm circumference; REE, resting energy expenditure; TSFT, triceps skinfold thickness.

Meanwhile, in CKD, malnutrition may worsen kidney disease and the glomerular filtration rate, which increases the risk of progression to end-stage renal disease and the start of peritoneal dialysis at a younger age. Additionally, a longer duration of dialysis is also associated with increased malnutrition [14]. The recommended energy intake for children with CKD stages 2–5 is around 100% of the estimated energy requirement for the chronological age, and adjustment is necessary according to each response, either weight gain or loss [47].

The typical diet for CKD should be include a restricted potassium intake of 40–120 mg/kg/d for infants and younger children, that of 30–40 mg/kg/d for older children, and a restricted sodium intake of 1500–2400 mg/d along with regular urine analysis and the timely introduction of transplant or dialysis service [48]. It has also been recently suggested that protein restriction is not recommended for children with CKD. The Kidney Disease Outcomes Quality Initiative advises 100–140% of the protein intake according to the Dietary Reference Intake for the ideal body weight in children with stage 3 CKD and 100–120% of protein intake in children with stages 4–5 CKD, because dialysis has been associated with further protein losses, which makes protein calculation for children undergoing dialysis crucial [47]. For infants with CKD, breastmilk is the optimal source of nutrition due to its low renal solute load. In cases where a mother is unable to provide adequate breastmilk, regular infant formula can be added accordingly [49].

CLD with malnutrition enhances the susceptibility to infection and increases morbidity and mortality risks. Due to malnutrition, patients suffer from diarrhea, bleeding, or bone disease. These also affect the outcomes of liver transplantation [10]. Children with CLD have increased energy needs, and the total energy intake should be increased to 140–200% of the estimated average requirements. Infants can benefit from breastfeeding, with a reduced risk of liver disease progression [50]. Moreover, some infants can be supplemented with medium-chain triglyceride (MCT) formulas, whereas older children can be given high-calorie, nutrient-dense drinks. Formula milk containing up to 75% fat as MCTs can be given; however, formula milk with MCTs above 80% can worsen steatorrhea and bring about essential fatty acid deficiency [51]. These can be given either orally or nasogastrically as required. The daily energy intake should cover 6–8 g/kg/d of carbohydrates, 2.5–3 g/kg/d of protein, and 5–6 g/kg/d of fat, and is calculated based on an increased calorie intake as mentioned earlier [52].

In addition, undernourishment in children with cancer reduces immunocompetence and causes delayed wound healing, decreased survival rates, prolonged hospital stays, increased risk of readmission, and reduced quality of life [41]. Nutritional assessment and intervention are important, especially in children with pre-existing malnutrition and at a high risk of nutrition depletion before cancer therapies. In all patients with cancer along with a functional gastrointestinal tract, nutrition should meet 95–100% of the estimated energy need [41,53]. It is suggested that arm anthropometry (TSFT and MUAC) gives the best estimate of the nutritional status, because these measurements are independent of tumor load and body weight can be deformed by cancer mass, organomegaly, ascites, or edema [53]. A study suggested the use of bioimpedance measurements for pediatric patients with a low BMI, which may give more information on muscle mass and nutritional risk [54].

Management should then be individualized according to each response. Breastmilk is optimal for infants with cancer, and if required, high-energy protein formulas can be offered to increase calorie density.

A balanced diet with adequate protein and high-energy levels is essential to avoid excessive carbohydrate and fat consumption, as noticed in children with cancer [41].

## Discussions

Consistent evidence suggests that Indonesia, like other developing countries, is very vulnerable to nondisease (or nonillness)–related malnutrition [55]. However, there are an increasing number of disease-related malnutrition cases, which requires more attention.

### Identifying malnutrition

In the literature review, we found some challenges in identifying malnutrition in chronic disease. First, we found that there was a lack of a uniform definition of malnutrition as a result of chronic disease, which made the literature search challenging. We retrieved articles that focused on nutrition-related chronic disease (such as diabetes and hypertension) and disease-related malnutrition while some centers used the term “illness-related malnutrition.”

Second, although the consensus regarding the definition of disease-related malnutrition requires a 3-mo duration [3], some studies diagnosed disease-related malnutrition at admission based on the complexity of disease and the length of stay [32,34] and it was not clear whether the patients were newly diagnosed with existing cachexia or readmitted with ongoing chronic disease; thus, these cases could be acute disease-related malnutrition, and there is the possibility of underdiagnosis of chronic disease-related malnutrition. In developing countries, the definition of malnutrition can be indistinct, not only based on the chronicity but also on the etiology. Some children may have been malnourished prior to disease or while experiencing disease-related malnutrition, and also have limited access to food, which may require more intensive management. The latter case may need access to appropriate nutritional supplement in addition to the underlying disease treatment.

### Anthropometric measurements and screening tools

For anthropometric parameters, in conditions such as CKD, CHD, CLD, and malignancy with altered body composition, some adjustments need to be made in choosing the right measurement. In hospitals where DXA is not readily available, the measurement can be performed with the closest anthropometry parameters based on studies that compared DXA using standardized protocols. For example, a study observed that the MUAC can provide a good estimate of lean body mass as measured by DXA in identifying the malnutrition risk in children with cancer [24].

There are several clinical tools developed for the identification of children at the risk of malnutrition, yet thus far, there is no consensus on the ideal screening tool to determine malnutrition risk in pediatric patients with chronic disease and there is no agreement between the screening tools. There are, thus far, no gold-standard methods for nutritional risk assessment. One way to consider the screening tools, especially in developing countries, is by checking their applicability in each hospital. The ease or difficulty of implementing each of these screening tools can be determined according to the availability of the healthcare team. PYMS and STAMP were, for example, developed for nurses; STRONGkids, on the other hand, has 2 questions that need to be answered by parents and the other 2 by healthcare professionals (doctors, pediatricians, dieticians, or nurses) [56]. Moreover, the PRNS tool requires 48 hours for the completion of the scores, whereas the STAMP and STRONGKids tools, when applied by trained assessors, took around 10–15 min and 5 min, respectively [57].

A study by Poulimeneas et al. [58] also highlighted the importance of malnutrition screening tools specific for each underlying disease, which considers the pathophysiology of the disease and other disease-related factors contributing to the development of malnutrition.

Ultimately, however, the applicability of anthropometric measurements and screening tools needs to be assessed by each center according to their feasibility.

## Conclusions

In developing countries, the prevalence of disease-related malnutrition in pediatric patients may be underestimated, especially in those with pre-existing malnutrition prior to disease development. The best screening tool for the identification of malnutrition risk is the one that is most applicable in each health unit inasmuch as there is systematic nutritional screening with regular follow-ups to identify the malnutrition risk in both inpatient and outpatient settings.

Nutrition is unquestionably a crucial aspect in children with chronic disease. It would be beneficial for tertiary hospitals to have a good network system with primary health care; thus, evaluation and regular nutritional assessment can be regularly and continuously evaluated. Although there is no uniform definition of malnutrition nor a consensus regarding screening tools, hospitals in developing countries need to focus on screening systems, professional teams, and management that work best according to the hospital’s own capacity.

## Acknowledgments

The authors’ responsibilities were as follows – YD, NN, RBK, BS, MA, IGLS: conceptualized and designed the study and critically revised the manuscript; MP, YD, NN, RBK, BS, MA, IGLS: acquired and analyzed the literatures used and drafted the manuscript; NLS, RB: critically revised the manuscript and provided final approval; and all authors: read and approved the final version of the manuscript.

## Author disclosures:

MP, YD, NN, RBK, BS, MA, and IGLS, no conflicts of interest. NLS and RWB, employees of Danone Specialized Nutrition Indonesia.

## Funding

The authors received no specific funding for this work.

## Data Availability

Data described in the manuscript will be made available pending application and approval.
